# Guided vs. conventional anti-platelet therapy for patients with acute coronary syndrome: A meta-analysis of randomized controlled trials

**DOI:** 10.3389/fcvm.2023.1079332

**Published:** 2023-03-21

**Authors:** Peng-Yu Zhong, Jian-Ping Deng, Jing-Hong Zhao, Lei Peng, Tao Liu, Hao-Yu Wang

**Affiliations:** ^1^ Department of Cardiology, Nanchong Central Hospital, The Second Clinical Medical College of North Sichuan Medical College, Nanchong, China; ^2^ Department of Urology, The Second Hospital of Lanzhou University Medical School, Lanzhou, China; ^3^ South China Hospital, Health Science Center, Shenzhen University, Shenzhen, China

**Keywords:** acute coronary syndrome, percutaneous coronary intervention, dual antiplatelet therapy, genotype testing, platelet function testing

## Abstract

**Background:**

Whether guided antiplatelet therapy in patients with acute coronary syndrome (ACS) is effective in improving net clinical benefits compared with conventional antiplatelet therapy remains controversial. Therefore, we assessed the safety and efficacy of guided antiplatelet therapy in patients with ACS and undergoing percutaneous coronary intervention.

**Method:**

We searched PubMed, EMBASE, and Cochrane Library databases to select the relevant randomized controlled trials comparing the guided and conventional antiplatelet therapy in patients with ACS. The primary and safety outcomes are major adverse cardiovascular events (MACE) and major bleeding, respectively. The efficacy outcomes included myocardial infarction, stent thrombosis, all-cause death, and cardiovascular death. We selected the relative risk (RR) and 95% confidence intervals (CIs) as effect size and calculated it using the Review Manager software. In addition, we evaluated the final results by trial sequential analysis (registered by PROSPERO, CRD 42020210912).

**Results:**

We selected seven randomized controlled trials and included 8,451 patients in this meta-analysis. Guided antiplatelet therapy can significantly reduce the risk of MACE (RR 0.64, 95% CI 0.54–0.76, *P* < 0.00001), myocardial infarction (RR 0.62, 95% CI 0.49–0.79, *P* = 0.0001), all-cause death (RR 0.61, 95% CI 0.44–0.85, *P* = 0.003), and cardiovascular death (RR 0.66, 0.49–0.90, *P* = 0.009). In addition, there is no significant difference between the two groups in stent thrombosis (RR 0.67, 95% CI 0.44–1.03, *P* = 0.07) and major bleeding (RR 0.86, 95% CI 0.65–1.13, *P* = 0.27). The subgroup analysis showed that the guided group based on genotype tests could bring benefits in MACE and myocardial infarction.

**Conclusions:**

The guided antiplatelet therapy is not only associated with a comparable risk of bleeding but also with a lower risk of MACE, myocardial infarction, all-cause death, cardiovascular death, and stent thrombosis than the conventional strategy in patients with ACS.

## Introduction

Dual antiplatelet therapy (DAPT) is a cornerstone for preventing ischemic complications in patients with acute coronary syndrome (ACS) and undergoing percutaneous coronary intervention (PCI) (1, 2). Clopidogrel is the most common type of P2Y_12_ receptor inhibitor, which needs to be converted to its active metabolite through cytochrome P450 (CYP2C19) enzymes in the liver (3, 4). However, there are individual differences in metabolic processes. In clinical practice, some patients could have high platelet reactivity (HPR) and/or CYP2C19 loss-of-function alleles, which are associated with higher thrombosis risk (4–6). On the contrary, the newer generation P2Y_12_ inhibitors, prasugrel and ticagrelor, have potent effects in inhibiting the aggregation of platelet and are not modulated by CYP2C19 genes (4, 7). Hence, the potent P2Y_12_ inhibitors could reduce the ischemic risk than clopidogrel in patients mentioned above, and clopidogrel could be fitter for patients without HPR or CYP2C19 loss-of-function alleles (8, 9). Meanwhile, there is higher risk of thrombosis in patients with ACS. Both the American Heart Association and European Society of Cardiology guidelines recommended that prasugrel or ticagrelor should be considered for patients with ACS (10, 11). However, the application of a potent P2Y_12_ inhibitor will increase the risk of bleeding and cause financial burden (8, 9). Therefore, genotype and platelet function testing have been two new potential approaches to choosing the optional P2Y_12_ inhibitor based on the individual difference.

A series of clinical trials have verified the efficacy and safety of shortening the duration of DAPT. Therefore, the 2017 European Society of Cardiology guideline recommended that patients with ACS should accept 12 months of DAPT, and the duration can be adjusted according to the risk of ischemia and bleeding (10, 11). However, the risk of ischemia and bleeding is hard to evaluate, and the individual antiplatelet strategy could be the best strategy for patients with ACS. Platelet function and genetic testing in patients after PCI represent two new schemes to guide antiplatelet therapy (4). More recent studies compared the guided and conventional strategies but have not provided an unequivocal result (12–14).

Therefore, we aim to explore the safety and efficacy of guided comparing conventional selection of antiplatelet therapy in patients with ACS. Related subgroup analysis was also performed to explore the impact of different strategies. In addition, trial sequential analysis (TSA) was used to assess the outcomes.

## Method

### Data source, inclusion, and exclusion criteria

We implemented this meta-analysis according to the Preferred Reporting Items for Systematic Review and Meta-Analysis guideline (15). We searched PubMed, EMBASE, and Cochrane Library databases for related randomized controlled trials, which compared guided antiplatelet therapy strategies (platelet function or genetic testing) with the conventional antiplatelet therapy. We also screened the abstracts of the scientific conferences in recent 3 years, such as the American Heart Association, American College of Cardiology, Transcatheter Cardiovascular Therapeutics, European Society of Cardiology, and Congress of the European Association of Percutaneous Cardiovascular Interventions. The major search terms in PubMed are as follows: “acute coronary syndrome” OR “percutaneous coronary intervention” AND “clopidogrel” OR “aspirin” OR “P2Y_12_ inhibitor” OR “prasugrel” OR “ticagrelor” AND “Randomized Controlled Trial”. The language was not limited in the process of literature retrieval and the detailed search strategy is shown (Supplementary Tables S1–S3).

The inclusion criteria of this meta-analysis met the following requirements: (a) patients with ACS and undergoing percutaneous coronary intervention; (b) compared guided and conventional antiplatelet therapy; (c) follow-up duration ≥6 months; (d) reported the efficacy and/or safety outcomes; and (e) randomized controlled trials. The exclusion criteria included: (a) reduplicate report and insufficient data from original studies and (b) nonrandomized controlled trial.

### Quality assessment, clinical outcomes, and data acquisition

We conducted quality assessment using the Cochrane tool of Collaboration. Meanwhile, Grades of Recommendations Assessment, Development and Evaluation (GRADE) was applied to evaluate the quality of each outcome (16, 17). The study was registered in PROSPERO (CRD42020210912). The primary outcome is major adverse cardiovascular events (MACE), which are composed of death, myocardial infarction, stroke, stent thrombosis, or bleeding. The efficacy outcomes included all-cause death, cardiovascular death, myocardial infarction, and definite or probable stent thrombosis. We selected major bleeding as the safety outcome, but there are different definitions of major bleeding in different trials. As a result of Bleeding Academic Research Consortium (BARC) 3 or 5 is same as TIMI minor or major, the major bleeding of this study mainly include those two types of bleeding.

The data from randomized controlled trials based on intention-to-treat analysis were extracted independently by two investigators (P-YZ and J-PD). We selected the relevant trials by screening initially all the titles and abstracts. After that, eligible trials were included by reviewing the full-text articles of the relevant studies. Any disagreement was solved through a discussion with a third party. In addition, the baseline characteristics were also independently extracted by two researchers, and the discrepancy was resolved through negotiation with one of the authors (TL).

### Statistical analysis

We performed the statistical analysis by Review Manager version 5.4 (Revman, The Cochrane Collaboration, Oxford, United Kingdom) and STATA 14.1 (StataCorp, College Station, TX, United States). The effect size is the relative risk (RR) with 95% confidence intervals (95% CI), which was calculated by the fixed-effects model based on the M-H statistical method. The *P* value of the chi-square test was used to evaluate heterogeneity, and the *I*^2^ index was employed to summarize the degree of heterogeneity. Significant heterogeneity was found, when the *P* value was <0.1 in comparison within groups or it was ≤0.05 in comparison among groups. The *I*^2^ values of 25%, 50%, and 75% correspond to low, moderate, and high heterogeneity, respectively (18). Subgroup analysis was performed according to (a) the different guided strategies and (b) de-escalation and escalation strategies. Publication bias was assessed by Egger's and Begg's tests, as well as the visual funnel plot. Trial sequential analysis version 0.9.5.10 software (Copenhagen Trial Unit, CTU) was applied to assess the results (available from https://www.ctu.dk/tsa).

## Results

### Search results and baseline characteristics

**Figure 1:**
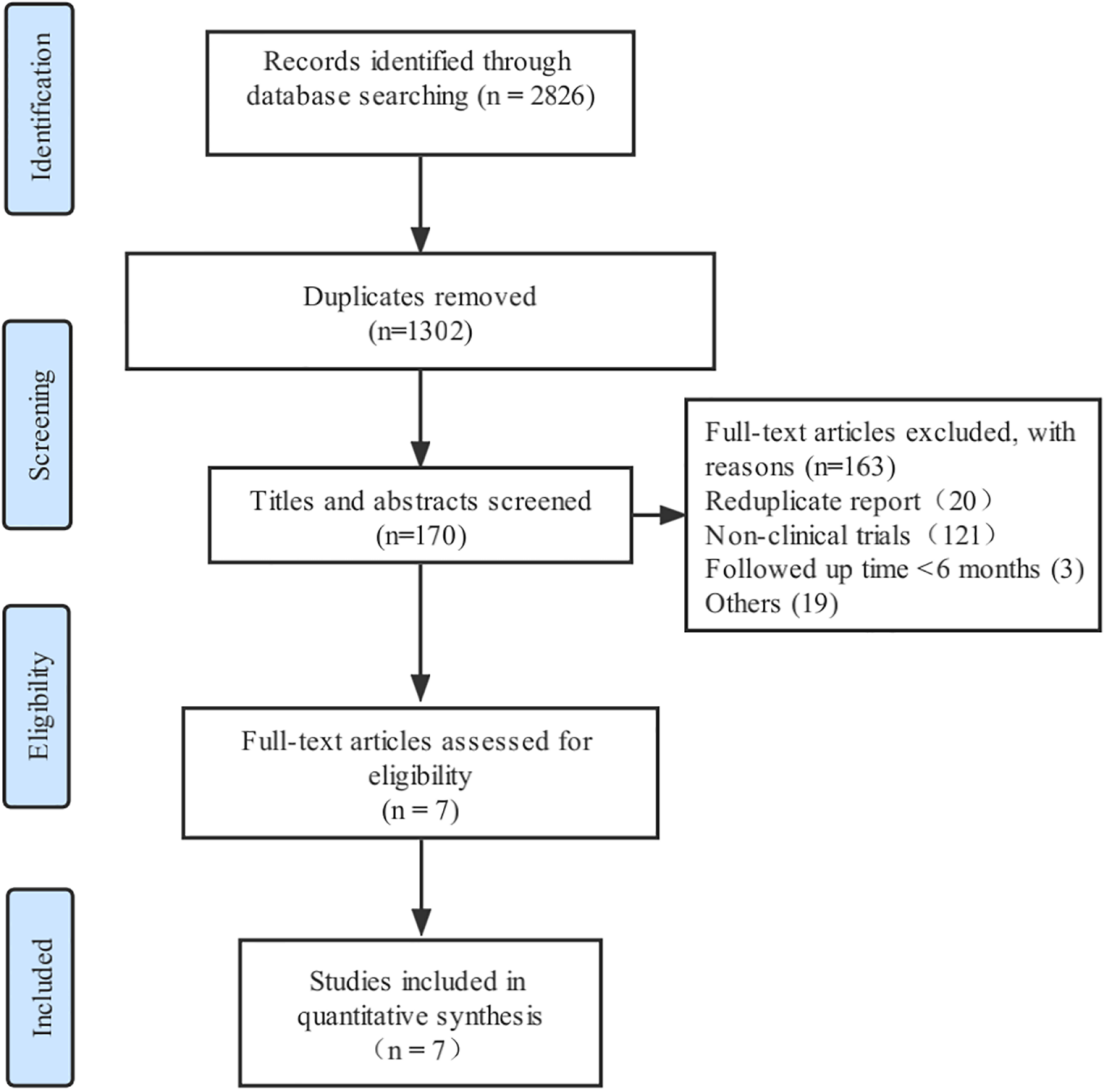
Flow diagram of literature search.

The process of literature screening and study selection is shown in Figure 1. A total of 2,826 randomized controlled trials were retrieved from PubMed, EMBASE, and Cochrane library databases. Finally, seven randomized controlled trials met the inclusion criteria after reviewing 27 full-text articles (19–25).

The characteristics of the included trials are shown (Table 1). Five and two randomized controlled trials applied genetic testing and platelet function testing, respectively. Meanwhile, this study included escalation and de-escalation strategies. The baseline characteristics of the patient are shown in Table 2. There were no significant distinctions in clinical presentations between the guided and conventional groups.

**Table 1:** Baseline characteristics of the included trials.

Trials	IAC-PCI	PHARMCLO	POPular Genetics	Shi et al.	Al-Rubaish et al.	ANTARCTIC	TROPICAL-ACS
Author	Xie	Notarangelo	Claassens	Shi	Al-Rubaish	Cayla	Sibbing
Year	2013	2018	2019	2021	2021	2016	2017
Design	Single center	Single center	Multicenter	Single center	Multicenter	Multicenter	Multicenter
Ethnicity	Chinese	European	European	Chinese	NR	European	European
Patients	ACS (100%)	ACS (97.2%)	STEMI (100%)	ACS (100%)	STEMI (100%)	ACS (100%)	ACS (100%)
Type of test	Genetic testing	Genetic testing	Genetic testing	Genetic testing	Genetic testing	Function testing	Function testing
**Guided group**
Clopidogrel	90%	43%	61%	42.8%	91%	39%	39%
Ticagrelor		43%	38%	57.2%	9%	59%	61%
Prasugrel		8%	1%				
**Conventional group**
Clopidogrel	100%	51%	90%	75%	100%	4%	
Ticagrelor		33%		25%		94%	100%
Prasugrel		8%					
Strategy	Escalation	—	De-escalation	Escalation	Escalation	De-escalation	De-escalation
Follow-up (months)	6	12	12	12	12	12	12

STEMI, ST-elevation myocardial infarction; ACS, non-ST-elevation acute coronary syndrome; NR, not reported.

**Table 2:** Baseline characteristics of the patients included.

Trials	IAC-PCI	PHARMCLO	POPular Genetics	Shi et al.	Al-Rubaish et al.	ANTARCTIC	TROPICAL-ACS
Age	57.9/57.8	71.1/70.7	61.9/61.4	59.7/59.8	56.7/55.5	80/81	59/58.5
Woman (%)	19.9/24.1	34.2/29.5	25.5/24	36.9/31	19.2/19.2	38/41	21/22
Diabetes (%)	30.6/32.4	25.2/27.7	11.1/11.1	37.3/38	89.4/84.4	28/28	18/22
Hypertension (%)	53.5/57.2	73.9/74.8	41/41	63.2/66	82.7/83.3	72/72	61/62
Active smoking (%)	NR	20.5/24.6	45.8/45.8	33/34	48.6/46.5	9/9	45/45
Dyslipidemia (%)	NR	56/52.7	20.5/20.5	68.7/67	86.1/83.8	53/55	42/41
Previous ACS (%)	NR	21.4/21.6	7.8/7	NR	54.4/54.7	19/15	11/12
Previous PCI (%)	NR	18.1/20	8/7.3	32.9/39	NR	24/26	13/14
Previous CABG (%)	NR	9.6/8.4	7/1.8	2/3	5.5/6.9	7/5	3/4

Data are shown as guided/conventional groups.

ACS, acute coronary syndrome; PCI, percutaneous coronary intervention; NR, not reported; CABG, coronary artery bypass grafting.

### The primary outcomes and subgroup analysis

**Figure 2:**
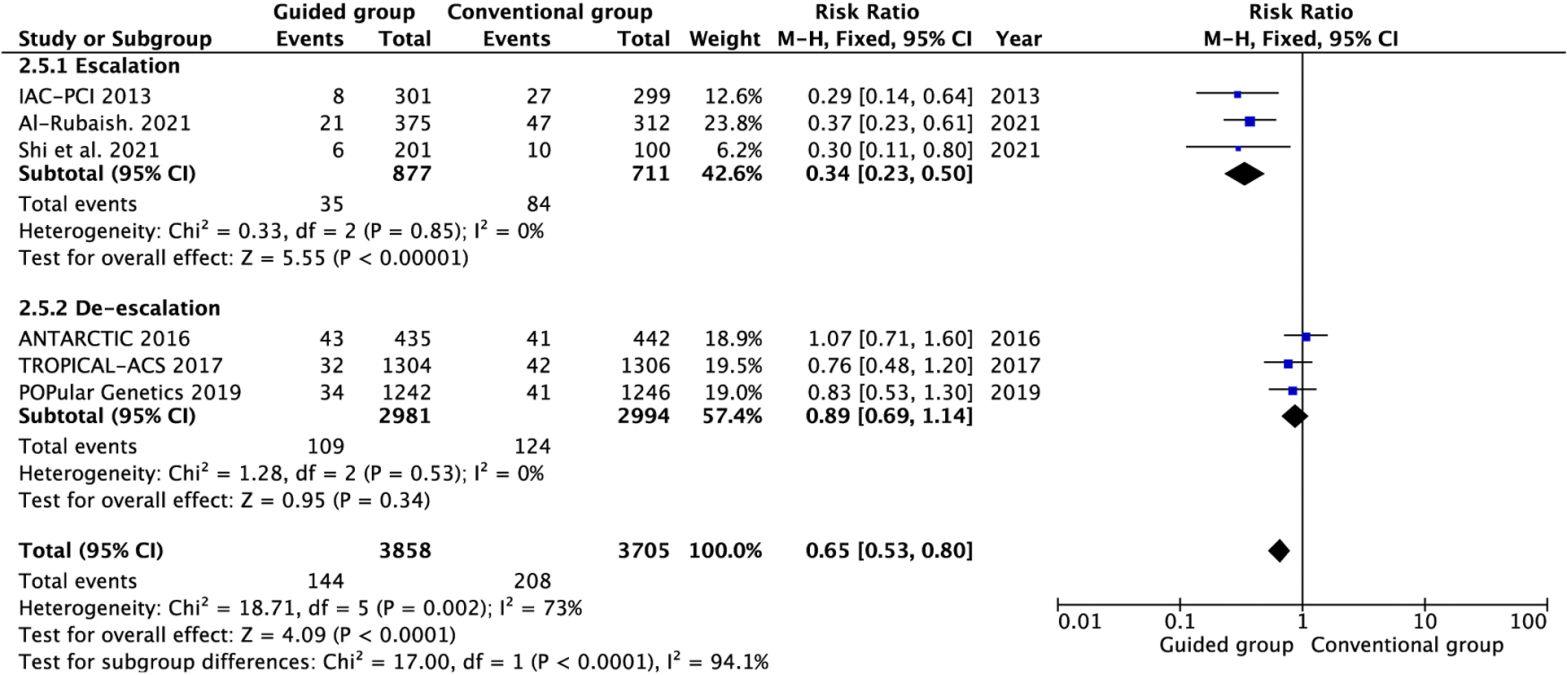
Pooled analyses of guided vs. standard antiplatelet therapy for the primary outcomes.

All included studies reported the incidence of MACE between the guided and conventional groups (Figure 2 and Supplementary Tables S2, S3). Compared with conventional group, the guided group can significantly reduce the incidence of MACE (RR 0.64, 95% CI 0.54–0.76, *P* < 0.00001, *I*^2^ = 68%, *P*_heterogeneity _= 0.004). There is statistical heterogeneity, and we conducted subgroup analyses to find the possible reason. This result is consistent in the escalation subgroup (RR 0.34, 95% CI 0.23–0.50, *P* < 0.00001, *I*^2^ = 0%, *P*_heterogeneity _= 0.85), but the incidence of MACE was not reduced in the guided group in de-escalation (RR 0.89, 95% CI 0.69–1.14, *P* = 0.34, *I*^2^ = 0%, *P*_heterogeneity _= 0.53). Meanwhile, there is significant statistical distinction between escalation and de-escalation subgroups (*P*_interaction_ < 0.00001). In addition, guided group based on genotype testing also can reduce the MACE risk (RR 0.54, 95% CI 0.44–0.66, *P* < 0.00001, *I*^2^ = 61%, *P*_heterogeneity_ = 0.04). However, this risk not decreased in the guided group based on platelet function (RR 0.91, 95% CI 0.67–1.23, *P* = 0.55, *I*^2^ = 14%, *P*_heterogeneity _= 0.28), and two subgroups have significant distinction (*P*_interaction _< 0.005). Similarly, there is a significant distinction in the genotype and platelet function testing subgroups (*P*_interaction _< 0.005).

### The efficacy outcomes and subgroup analysis

**Figure 3:**
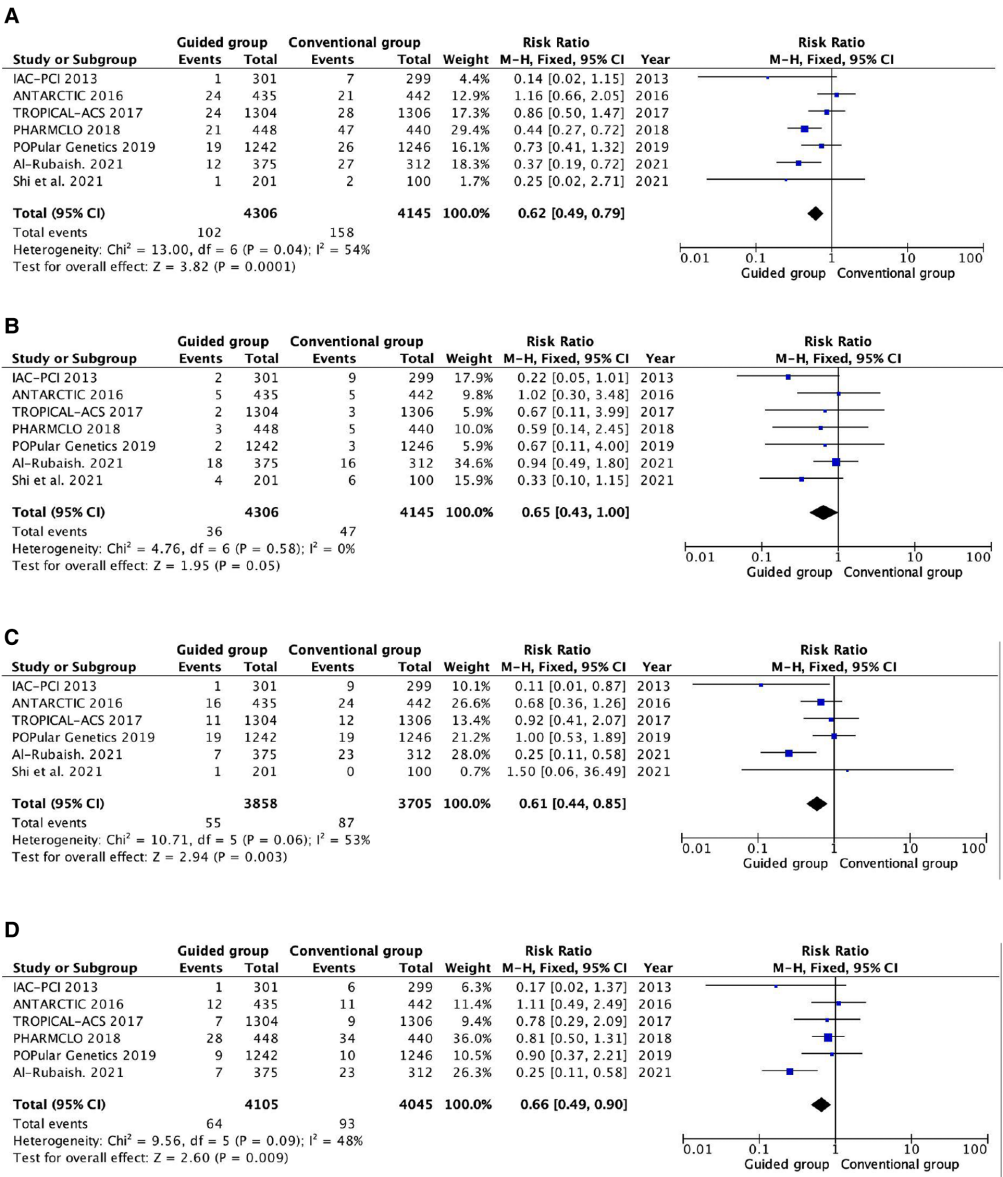
Pooled analyses of guided vs. standard antiplatelet therapy for the efficacy outcomes. (**A**) Myocardial infarction; (**B**) stent thrombosis; (**C**) all-cause death; (**D**) cardiovascular death.

All the trails reported myocardial infarction and stent thrombosis (Figure 3). Compared with the conventional antiplatelet therapy, the guided group had a significantly reduced risk of myocardial infarction (RR 0.62, 95% CI 0.49–0.79, *P* = 0.0001, *I*^2^ = 54%, *P*_heterogeneity _= 0.04). On the contrary, there are no significant differences between the two groups in stent thrombosis (RR 0.65, 95% CI 0.43–1.00, *P* = 0.05, *I*^2^ = 0%, *P*_heterogeneity _= 0.58). The all-cause death and cardiovascular death are reported in six and five trials, respectively (Figure 3). The guided group is associated with a lower risk of all-cause death than the conventional group (RR 0.61, 95% CI 0.44–0.85, *P* = 0.003, *I*^2^ = 53%, *P*_heterogeneity_ = 0.06). Similarly, the guided group also has a lower risk of cardiovascular death (RR 0.66, 95% CI 0.49–0.90, *P* = 0.009, *I*^2^ = 48%, *P*_heterogeneity_ = 0.09) than the conventional group.

High heterogeneity was found in myocardial infarction, all-cause death, and cardiovascular death. We conducted two subgroup analyses to find the possible reason, and the results are shown in Supplementary Tables S2, S3. The distinction of the guided groups based on escalation and de-escalation strategies could be the most reasonable cause for the heterogeneity. In the escalation subgroup, no heterogeneity was found and the guided group had a significantly reduced incidence of myocardial infarction (RR 0.32, 95% CI 0.17–0.59, *P* = 0.0002, *I*^2^ = 0%, *P*_heterogeneity_ = 0.67), all-cause death (RR 0.24, 95% CI 0.12–0.50, *P* = 0.0001, *I*^2^ = 0%, *P*_heterogeneity_ = 0.40), and cardiovascular death (RR 0.24, 95% CI 0.11–0.51, *P* = 0.0003, *I*^2^ = 0%, *P*_heterogeneity_ = 0.71). However, there is no significant distinction between guided and conventional groups for those outcomes in the de-escalation subgroup.

In addition, the difference between guided antiplatelet therapy based on genotype and platelet function testing could be also another reason for heterogeneity. In the genotype testing subgroup, heterogeneity could be significantly reduced. The guided group is associated with a lower risk of myocardial infarction (RR 0.47, 95% CI 0.34–0.64, *P* < 0.00001, *I*^2^ = 8%, *P*_heterogeneity _= 0.36), cardiovascular death (RR 0.24, 95% CI 0.11–0.51, *P* = 0.0003, *I*^2^ = 0%, *P*_heterogeneity _= 0.7), and stent thrombosis (RR 0.61, 95% CI 0.38–0.98, *P* = 0.04, *I*^2^ = 7%, *P*_heterogeneity _= 0.37). On the contrary, the guided group has a similar risk of myocardial infarction, cardiovascular death, and stent thrombosis compared to the conventional group in the platelet function testing.

### The safety outcomes and subgroup analysis

**Figure 4:**
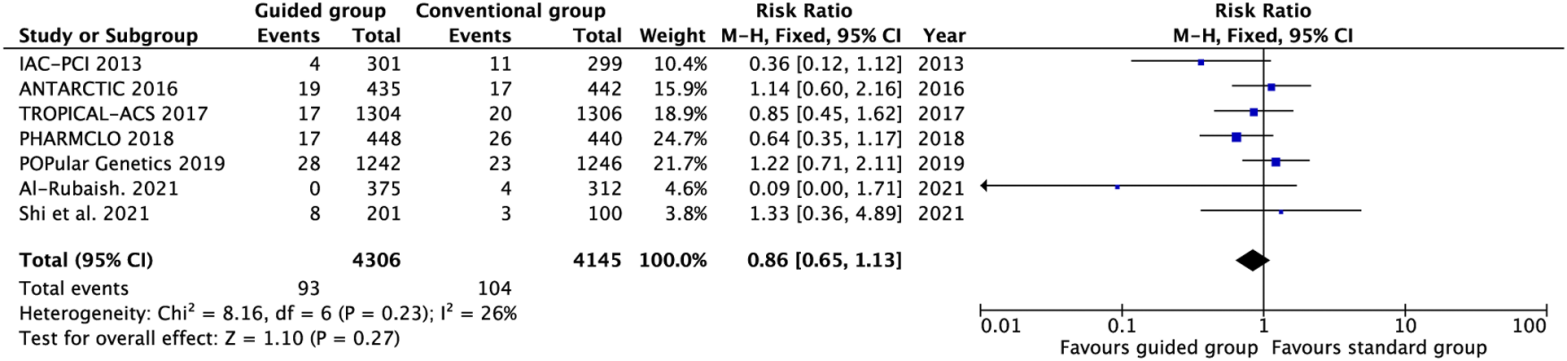
Pooled analyses of guided vs. conventional antiplatelet therapy for the safety outcomes.

All the trials reported the incidence of major bleeding (Figure 4 and Supplementary Tables S2, S3). There were no significant differences and heterogeneity in major bleeding between the guided and conventional groups (RR 0.86, 0.65–1.13, *P* = 0.27, *I*^2^ = 23%, *P*_heterogeneity_ = 0.26). The subgroup analysis suggested that no difference was found between escalation and de-escalation subgroups. Meanwhile, the different types of testing also did not impact the risk of major bleeding.

### Assessment of quality, trial sequential analysis, and publication bias

The quality assessment of each trial and the quality assessment of GRADE evidence are shown in the supplementary materials (Supplementary Figure S1 and Table S4). There is a low risk of bias in selection, detection, and reporting, but there is a high risk of bias in performance in three out of seven trials. The quality assessments of GRADE evidence of major bleeding is moderate, and other outcomes are high. The trial sequential analysis of each outcome was performed, and the results are shown in Supplementary Figure S2. The curves of MACE and myocardial infarction are beyond the TSA boundary. All-cause death and cardiovascular death were beyond the conventional boundary but did not reach the TSA boundary and met the expected sample size. The curves of stent thrombosis and major bleeding did not reach both the conventional boundary and the expected sample size. The expected sample size of both stent thrombosis and major bleeding is 19,606. The publication bias evaluation of each outcome shows that the spots of the funnel plot were symmetrically distributed, and the *P* values of Begg's and Egger's tests are more than 0.05 (Supplementary Figure S3 and Table S5).

## Discussion

The results of this meta-analysis were based on seven randomized controlled trials and 8,451 patients. The results showed that the guided group is associated with similar efficacy and lower safety than the conventional group. In addition, the guided group based on escalation strategy has a significantly reduced incidence of ischemic events and primary outcomes. Guided groups based on genotype testing and escalation strategies are associated with a lower risk of MACE and myocardial infarction.

The DAPT is effective in inhibiting platelet reactivity and preventing ischemic events in patients with ACS after PCI (26). However, it is doubtful whether guided DAPT can improve efficacy while decreasing bleeding risk. Ticlopidine, the first-generation P2Y_12_ inhibitor, was launched in the 1990s, but it was linked with several severe side effects (27). Aspirin combined with clopidogrel can reduce the risk of thrombotic events while maintaining an acceptable safety profile compared to aspirin monotherapy, which has been widely used in clinical practice. However, unlike the potent P2Y_12_ inhibitors, there are large individual differences in the metabolism process of clopidogrel, which may result in severe HPR. Furthermore, HPR is linked to thrombosis, which can be caused by a variety of conditions including age, BMI, chronic renal disease, and diabetes. In addition, as potent P2Y_12_ inhibitors, ticagrelor and prasugrel can further reduce the risk of ischemia than clopidogrel, but they bring higher risk of bleeding simultaneously (9). Therefore, potent P2Y_12_ receptor inhibitors can bring more ischemic benefit than clopidogrel in patients with HPR or CYP2C19 loss-of-function alleles. Based on the above-mentioned rationale, platelet function and genetic testing were applied to select the best antiplatelet agent to achieve individual therapy (4, 7, 28).

Galli et al. (29) performed a meta-analysis to compare the efficacy and safety of the guided and conventional groups. The results showed that guided antiplatelet therapy was associated with a lower incidence of MACE (RR 0.78, 95% CI 0.63–0.95, *P* = 0.015), cardiovascular death (RR 0.77, 95% CI 0.59–1.00, *P* = 0.049), myocardial infarction (RR 0.76, 95% CI 0.60–0.96, *P* = 0·021), and stent thrombosis (RR 0.64, 95% CI 0.46–0.89, *P* = 0.011). Meanwhile, the guided strategy had a lower bleeding risk but was not statistically significant (RR 0.88, 95% CI 0.77–1.01, *P* = 0.069). Our study is consistent with this meta-analysis. The subgroup analysis of this study showed that the escalation approach was associated with a significant reduction in ischemic events without any trade-off in safety, and the de-escalation approach was associated with a significant reduction in bleeding events, without any trade-off in efficacy.

A series of factors will affect the conclusions in clinical practice. At first, East Asians could be associated with higher bleeding risk and lower ischemic risk than Caucasians (30). Therefore, short-term DAPT composed of aspirin and clopidogrel could be fitter for them. However, the incidence of clopidogrel hyporesponsiveness in East Asian patients was higher than that in Caucasians, which may be due to their unique cytochrome gene polymorphism (30, 31). In addition, East Asians have a high rate of CYP2C19 loss-of-function alleles, and guided antiplatelet therapy could be more suited for East Asian patients. Second, although potent P2Y_12_ inhibitors (prasugrel or ticagrelor) are recommended in patients with ACS, clopidogrel is still applied in clinical practice, especially for old patients. There are significant differences between escalation and de-escalation strategies. Compared with clopidogrel, the guided strategy of escalation aims to increase efficacy by switching from clopidogrel to prasugrel or ticagrelor. Our study suggested that the guided group is correlated with higher efficacy than clopidogrel. Furthermore, the guided strategy would result in de-escalation, which is associated with lower risk of bleeding and without compromising efficacy. Our result is correlated with the recommendations, which showed a guided approach with compromising efficacy but not reducing the risk of bleeding.

At last, both genetic and platelet tests have superiority and inferiority in clinical practice. By characterizing the platelet phenotype, platelet function tests could better relate to thrombosis (32). However, platelet function tests require patients to be treated with clopidogrel to determine responsiveness and escalate to a more potent P2Y_12_ inhibitor when patients have HPR (32). In addition, there are variabilities in the test results. Therefore, it is a challenge to implement platelet function test monitoring in nonspecialized centers (33). On the contrary, genetic testing for CYP2C19 loss-of-function alleles can now be achieved by rapid bedside testing, which can overcome some of the limitations mentioned above (32). However, multiple factors can influence the effectiveness of antiplatelet agents including age, body mass index, and chronic kidney disease. Therefore, assessing only genetic polymorphisms may have limited accuracy in identifying patients with HPR status. Combining multiple clinical variables with genotype to predict HPR status is considered to have greater accuracy than the individual components.

### Limitations

This meta-analysis of randomized control trials has several limitations. First, this meta-analysis is based on study-level data and not based on the individual data. The majority of included trials are open-label, which may result in the risk of bias. Second, there are many distinctions in the baseline characteristics of included randomized controlled trials. For example, there are different rates of patients with ST-elevation myocardial infarction and durations of follow-up. Third, only two included trials researched the guided strategy based on platelet function, which does not bring clinical benefits for patients with ACS. Hence, the efficacy of platelet function needs to be tested by more trials in the future. Finally, there is high heterogeneity in primary and efficacy outcomes, and we have found possible reasons by subgroup analysis. Therefore, the main conclusions are stated by the results of the subgroup analysis, which included the limited number of patients and the need for a larger sample of randomized control trials.

## Conclusions

This systematic review and meta-analysis demonstrates that guided antiplatelet therapy after PCI was associated with a lower risk of MACE, myocardial infarction, all-cause death, and cardiovascular death in patients with ACS. Meanwhile, the guided strategy based on genotype testing could reduce the risk of MACE and myocardial infraction. At last, the guided group based on escalation strategy did not have increased risk of bleeding and improved the net clinical benefit, but the guided group based on the de-escalation strategy did not obtain net clinical benefits for patients with ACS.

## Data Availability

The original contributions presented in the study are included in the article/Supplementary Material, further inquiries can be directed to the corresponding authors.
