# Supplementary material for: Guided vs. conventional anti-platelet therapy for patients with acute coronary syndrome: A meta-analysis of randomized controlled trials
Source: Front Cardiovasc Med. 2023 Mar 21;10:1079332. doi: 10.3389/fcvm.2023.1079332 (PMC10070678; doi:10.3389/fcvm.2023.1079332)
Supplement: Supplementary file 1 [file Datasheet1.pdf]

Supplementary

Supplementary Table 1. The search strategies of three databases.

Database	Search strategies	Results
PubMed	((((((((((((genotype) OR (polymorphism)) OR (pharmacogenetic)) OR (pharmacogenomic)) OR (genetic)) OR (genomic)) OR (genotyping)) OR (variant)) OR (variation)) OR (cyp2c19)) OR (cytochrome p450 2c19)) AND (((((((((guide) OR (personalized)) OR (guided)) OR (guiding)) OR (tailored)) OR (individualized)) OR (individualizing)) OR (individualization)) OR (directed)) OR (directing))) AND ((((((((antiplatelet) OR (antithrombosis)) OR (clopidogrel)) OR (Iscover)) OR (Plavix)) OR (ticagrelor)) OR (prasugrel)) OR (thienopyridine)) OR (P2Y12 inhibitors))) AND ((((((Acute Coronary Syndromes) OR (ACS)) OR (Percutaneous Coronary Interventions)) OR (PCI)) OR (Percutaneous Coronary Revascularizations)) OR (Coronary Intervention))	1023
EMBASE	#1. 'genotype'/exp OR genotype OR 'polymorphism'/exp OR polymorphism OR pharmacogenetic OR pharmacogenomic OR 'genetic'/exp OR genetic OR genomic OR 'genotyping'/exp OR genotyping OR 'variant'/exp OR variant OR 'variation'/exp OR variation OR 'cyp2c19'/exp OR cyp2c19 OR 'cytochrome p450 2c19'/exp OR 'cytochrome p450 2c19' OR (('cytochrome'/exp OR cytochrome) AND ('p450'/exp OR p450) AND 2c19)<br>#2. 'guide'/exp OR guide OR personalized OR guided OR guiding OR tailored OR individualized OR individualizing OR 'individualization'/exp OR individualization OR directed OR directing<br>#3. antiplatelet OR 'antithrombosis'/exp OR antithrombosis OR 'clopidogrel'/exp OR clopidogrel OR 'iscover'/exp OR iscover OR 'plavix'/exp OR plavix OR 'ticagrelor'/exp OR ticagrelor OR 'prasugrel'/exp OR prasugrel OR 'thienopyridine'/exp OR thienopyridine OR 'p2y12 inhibitors' OR (p2y12 AND ('inhibitors'/exp OR inhibitors))<br>#4. 'acute coronary syndromes'/exp OR 'acute coronary syndromes' OR (acute AND coronary AND syndromes) OR 'acs'/exp OR acs OR 'percutaneous coronar<br>interventions' OR (percutaneous AND coronary AND ('interventions'/exp OR interventions)) OR pci OR 'percutaneous coronary revascularizations' OR (percutaneous AND coronary AND revascularizations) OR 'coronary intervention' OR (coronary AND ('intervention'/exp OR intervention))	972

	#5. #1 AND #2 AND #3 AND #4	
Cochrane library	#1 MeSH descriptor: [Genotype] explode all trees<br>#2 genotype OR polymorphism OR pharmacogenetic OR pharmacogenomic OR genetic OR genomic OR genotyping OR variant OR variation OR cyp2c19 OR<br>cytochrome p450 2c19<br>#3 MeSH descriptor: [Platelet Aggregation Inhibitors] explode all trees<br>#4 antiplatelet OR antithrombosis OR clopidogrel OR Iscover OR Plavix OR ticagrelor OR prasugrel OR thienopyridine OR P2Y12 inhibitors OR Platelet Aggregation Inhibitors<br>#5 Acute Coronary Syndromes OR ACS OR Percutaneous Coronary Interventions OR PCI<br>#6 guide OR personalized OR guided OR guiding OR tailored<br>OR individualized OR individualizing OR individualization<br>OR directed OR directing<br>#7 (#1 OR #2) AND (#3 OR #4) AND #5 AND #6	831

Supplementary Table 2. The subgroup analysis of guided group based on escalation and de-escalation strategies.

Outcomes		*Trials*	Heterogeneity		Effect Size			P interaction
			I^2 (%)	*P value*	*RR* (95%*CI*)	*P value*	I^2(%)	
MACE	Escalation	3	0	0.85	0.34(0.23,0.50)	<0.00001	94.1	<0.0001
	De-escalation	3	0	0.53	0.89(0.69,1.14)	0.34		
Myocardial infarction	Escalation	3	0	0.67	0.32(0.17,0.59)	0.0002	88.4	0.003
	De-escalation	3	0	0.53	0.90(0.65,1.24)	0.52		
All-cause death	Escalation	3	0	0.40	0.24(0.12,0.50)	0.0001	88.7	0.003
	De-escalation	3	0	0.67	0.84(0.57,1.24)	0.39		
Cardiovascular death	Escalation	2	0	0.71	0.24(0.11,0.51)	0.0003	88.3	0.004
	De-escalation	3	0	0.86	0.94(0.56,1.57)	0.81		
Stent thrombosis	Escalation	3	53	0.12	0.61(0.36,1.03)	0.06	0	0.56
	De-escalation	3	0	0.90	0.83(0.34,1.99)	0.67		
Major bleeding	Escalation	3	47	0.15	0.49(0.23,1.04)	0.69	70.7	0.06
	De-escalation	3	0	0.69	1.07(0.76,1.52)	0.69		

Supplementary Table 3. The subgroup analysis of guided group based on genotype and platelet function strategies.

Outcomes		*Trials*	Heterogeneity		Effect Size		I^2(%)	$P_{interaction}$
			I^2 (%)	*P value*	*RR* (95%*CI*)	*P value*		
MACE	Genotype	5	61	0.04	0.54(0.44,0.66)	<0.00001		0.005
	Platelet function	2	14	0.28	0.91(0.67,1.23)	0.55	80	
Myocardial infarction	Platelet function	2	0	0.45	0.99(0.67,1.46)	0.95		0.003
	Genotype	5	8	0.36	0.47(0.34,0.64)	<0.00001	88.3	
All-cause death	Genotype	4	0	0.56	0.76(0.46,1.24)	0.27		0.24
	Platelet function	2	69	0.02	0.51(0.32,0.80)	0.003	14	
Cardiovascular death	Genotype	4	62	0.05	0.58(0.41,0.84)	0.004		0.18
	Platelet function	2	0	0.59	0.96(0.52,1.79)	0.9	45.1	
Stent thrombosis	Platelet function	2	0	0.70	0.88(0.32,2.43)	0.81		0.51
	Genotype	5	7	0.37	0.61(0.38,0.98)	0.04	0	
Major bleeding	Platelet function	2	0	0.53	0.98(0.62,1.54)	0.94		0.46
	Genotype	5	46	0.12	0.79(0.56,1.12)	0.19	0	

Supplemental Table. 4 Quality assessments of GRADE evidence for each outcome.

Certainty assessment							Summary of findings				
Participants (studies) Follow-up	Risk of bias	Inconsistency	Indirectness	Imprecision	Publication bias	Overall certainty of evidence	Study event rates (%)		Relative effect (95% CI)	Anticipated absolute effects	
							With Conventional group	With Guided group		Risk with Conventional group	Risk difference with Guided group
**MACE**											
8451 (7 RCTs)	not serious	not serious	not serious	not serious	none	⨁⨁⨁⨁ High	302/4145 (7.3%)	202/4306 (4.7%)	**RR 0.64** (0.54 to 0.76)	73 per 1,000	**26 fewer per 1,000** (from 34 fewer to 17 fewer)
**Myocardial infarction**											
8451 (7 RCTs)	not serious	not serious	not serious	not serious	none	⨁⨁⨁⨁ High	158/4145 (3.8%)	102/4306 (2.4%)	**RR 0.62** (0.49 to 0.79)	38 per 1,000	**14 fewer per 1,000** (from 19 fewer to 8 fewer)
**Stent thrombosis**											
8451 (7 RCTs)	not serious	not serious	not serious	not serious	none	⨁⨁⨁⨁ High	47/4145 (1.1%)	36/4306 (0.8%)	**RR 0.65** (0.43 to 1.00)	11 per 1,000	**4 fewer per 1,000** (from 6 fewer to 0 fewer)
**All-cause death**											
7563 (6 RCTs)	not serious	not serious	not serious	not serious	none	⨁⨁⨁⨁ High	87/3705 (2.3%)	55/3858 (1.4%)	**RR 0.61** (0.44 to 0.85)	23 per 1,000	**9 fewer per 1,000** (from 13 fewer to 4 fewer)
**Cardiovascular death**											
8150 (6 RCTs)	not serious	not serious	not serious	not serious	none	⨁⨁⨁⨁ High	93/4045 (2.3%)	64/4105 (1.6%)	**RR 0.66** (0.49 to 0.90)	23 per 1,000	**8 fewer per 1,000** (from 12 fewer to 2 fewer)
**Major bleeding**											
8451 (7 RCTs)	not serious	not serious	not serious	serious	none	⨁⨁⨁◯ Moderate	104/4145 (2.5%)	93/4306 (2.2%)	**RR 0.86** (0.65 to 1.13)	25 per 1,000	**4 fewer per 1,000** (from 9 fewer to 3 more)

CI: confidence interval; **RR:** risk ratio

Supplementary Table 5. The Begg's and Egger's Test in STATA of each endpoints.

Endpoints	*P* (Begg's test)	*P* (Eegg's test)
Myocardial infarction	1.000	0.493
Stent thrombosis	0.734	0.224
All-cause death	0.734	0.440
Cardiovascular death	0.308	0.280
Major bleeding	1.000	0.509
MACCE	1.000	0.908

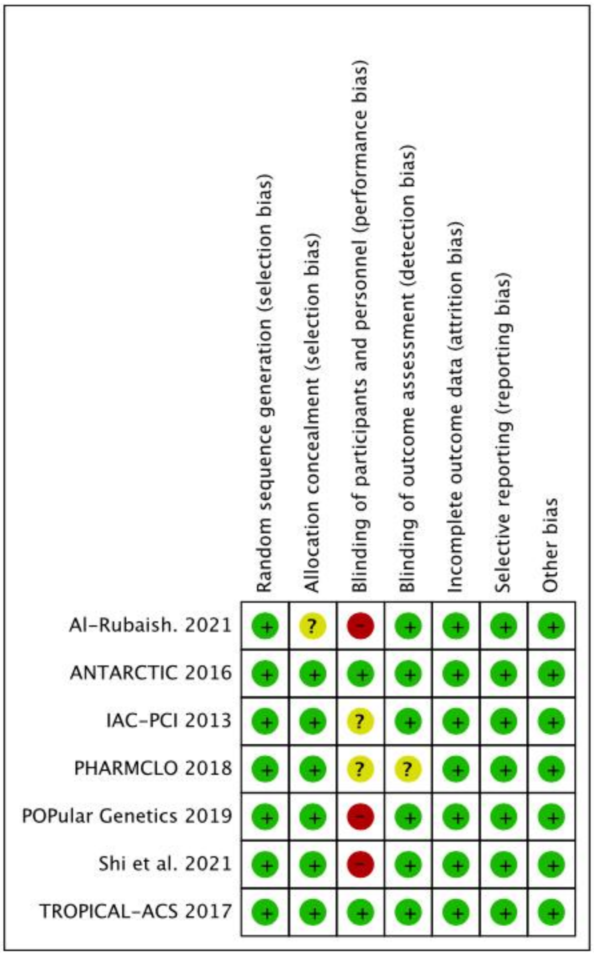

Supplementary Figure 1. Bias risk assessment of the studies.

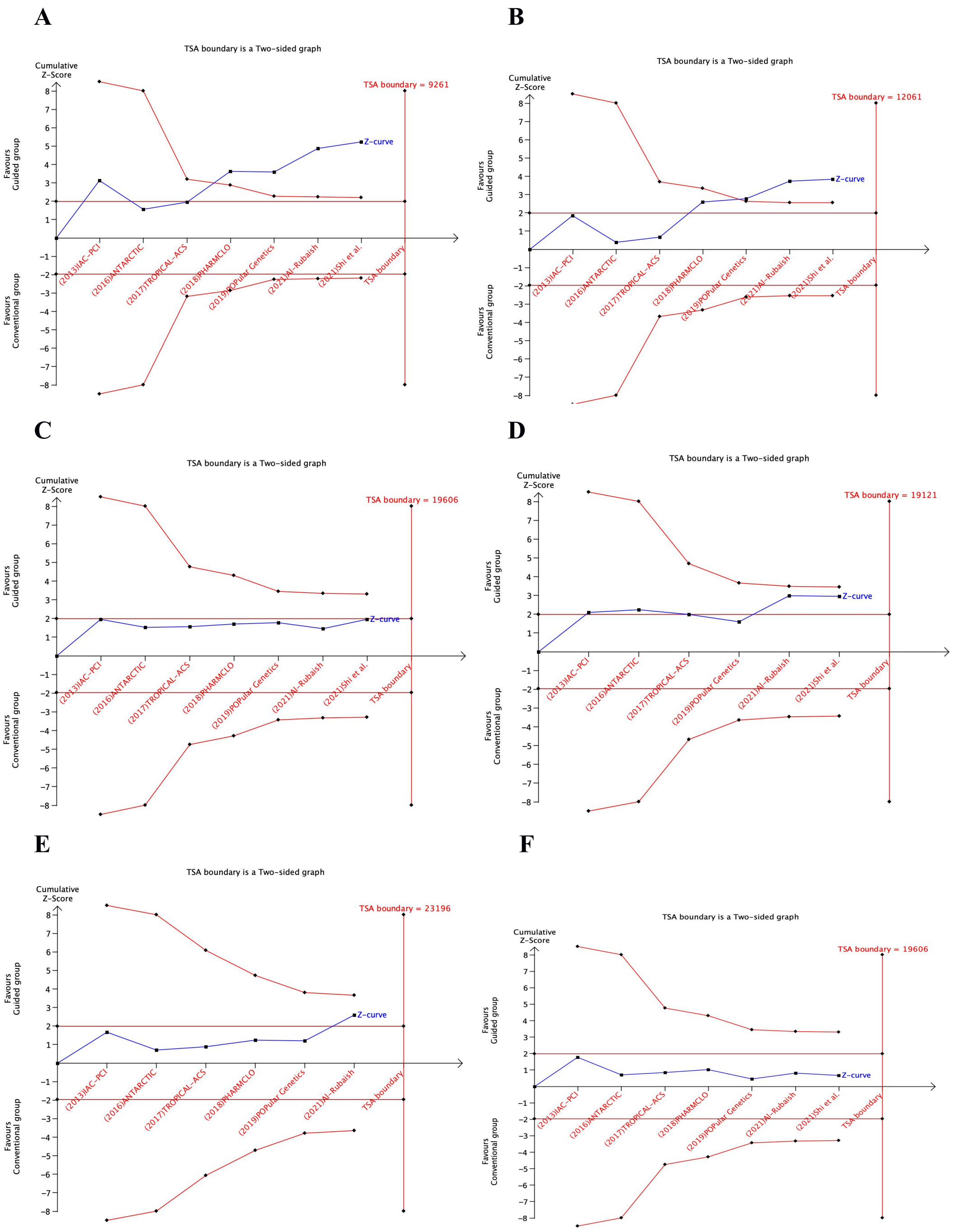

Supplemental Figure 2. The results of TSA between guided and conventional groups. (A) MACE, (B) myocardial infarction, (C) stent thrombosis, (D) all-cause death, (E) cardiovascular death, (F) Major bleeding.

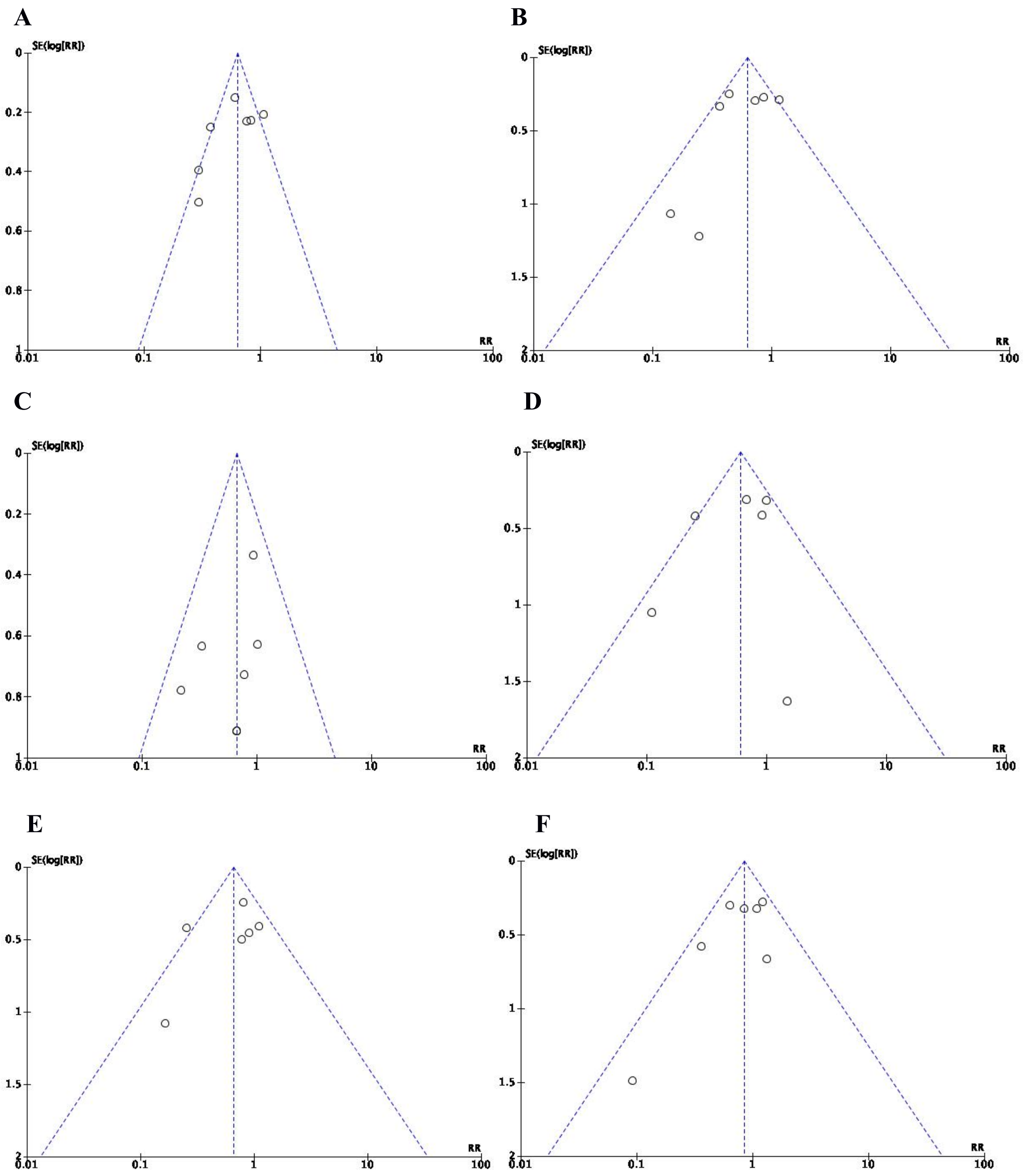

Supplemental Figure 3. The funnel plots of every outcomes. (A) MACE, (B) myocardial infarction, (C) stent thrombosis, (D) all-cause death, (E) cardiovascular death, (F) Major bleeding.
